# CRISPR-Cas9-Mediated ATF6B Gene Editing Enhances Membrane Protein Production in HEK293T Cells

**DOI:** 10.3390/bioengineering12040409

**Published:** 2025-04-11

**Authors:** Ho Joong Choi, Ba Reum Kim, Ok-Hee Kim, Say-June Kim

**Affiliations:** 1 Department of Surgery, Division of Hepatobiliary Pancreatic Surgery, Seoul St. Mary’s Hospital, College of Medicine, The Catholic University of Korea, 222, Banpo-daero, Seocho-gu, Seoul 06591, Republic of Korea; hopej0126@catholic.ac.kr; 2 Catholic Central Laboratory of Surgery, College of Medicine, The Catholic University of Korea, Seoul 06591, Republic of Korea; ok6201@hanmail.net; 3 Translational Research Team, Surginex Co., Ltd., Seoul 06591, Republic of Korea; brkim@surginex.com

**Keywords:** CRISPR-Cas9, gene editing, HEK293T cells, ATF6B editing, membrane protein production, unfolded protein response

## Abstract

This study aims to enhance membrane protein production in HEK293T cells through genetic modification. HEK293T cells are used for recombinant protein and viral vector production due to their human origin and post-translational modification capabilities. This study explores enhancing membrane protein production in these cells by deleting the C-terminal of the ATF6B gene using CRISPR-Cas9 technology. The objective of this research is to investigate the effect of C-terminal deletion of the ATF6B gene on membrane protein production in HEK293T cells using CRISPR-Cas9 technology. To identify effective gene targets, sgRNAs were initially designed against multiple UPR-related genes, including ATF6A, IRE1A, IRE1B, PERK, and ATF6B. Among them, ATF6B was selected as the primary target for further investigation due to its superior editing efficiency. The efficiency of sgRNAs was evaluated using the T7E1 assay, and sequencing was performed to verify gene editing patterns. Membrane proteins were extracted from both ATF6B C-terminally deleted (ATF6B-ΔC) and wild-type (WT) cell lines for comparison. Flow cytometry was employed to assess membrane protein production by analyzing GFP expression in Membrane-GFP-expressing cells. HEK293T cells with C-terminally deleted ATF6B (ATF6B-ΔC) significantly increased membrane protein production by approximately 40 ± 17.6% compared to WT cells (*p* < 0.05). Sequencing revealed 11, 14, 1, and 10 bp deletions in the ATF6B-ΔC edited cells, which disrupted exon sequences, induced exon skipping, and introduced premature stop codons, suppressing normal protein expression. Flow cytometry confirmed a 23.9 ± 4.2% increase in GFP intensity in ATF6B-ΔC cells, corroborating the enhanced membrane protein production. These findings suggest that CRISPR-Cas9-mediated C-terminal deletion of the ATF6B gene can effectively enhance membrane protein production in HEK293T cells by activating the unfolded protein response pathway and improving the cell’s capacity to manage misfolded proteins. This strategy presents significant potential for the biotechnology and pharmaceutical industries, where efficient membrane protein production is essential for drug development and various applications.

## 1. Introduction

The unfolded protein response (UPR) is an essential cellular mechanism activated in response to the accumulation of unfolded or misfolded proteins in the endoplasmic reticulum (ER) [1,2,3]. This process is critical for maintaining cellular homeostasis and proper protein folding. The UPR is primarily regulated by three ER stress sensors: ATF6, IRE1, and PERK [1,3]. Among these, ATF6, which includes ATF6A and ATF6B isoforms, plays a central role by translocating to the Golgi upon ER stress, where its active form is generated to initiate transcriptional responses [4,5]. Among the UPR regulators, ATF6B has emerged as a potential target for modulating membrane protein production due to its distinct role in ER stress adaptation.

**Figure 1:**
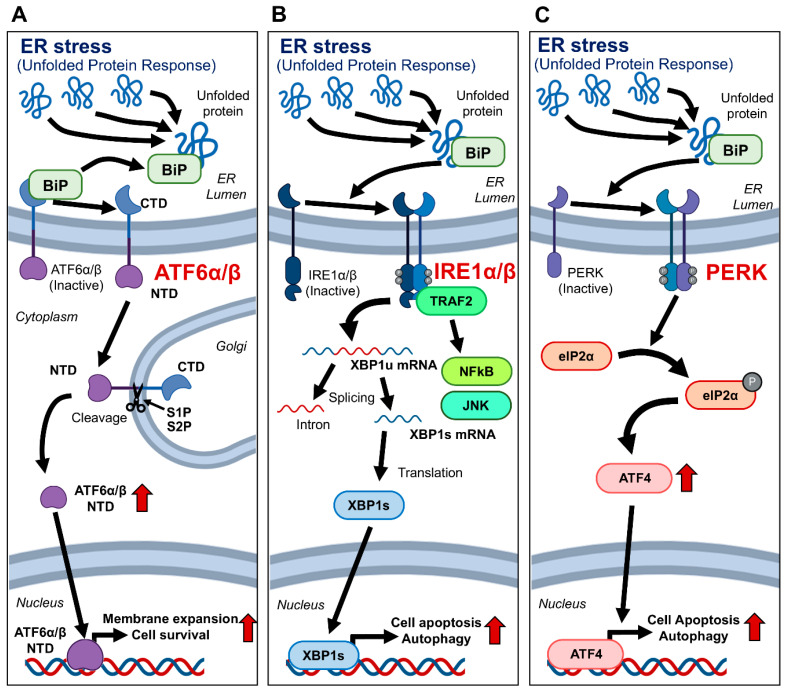
Signal transduction pathways of major regulatory candidate genes in the UPR process. Schematic representation of the signal transduction pathways involving candidate genes that play major regulatory roles in the UPR process. (**A**) ATF6α/β is typically located in the ER membrane under normal conditions. Upon stimulation by unfolded proteins, it translocates to the Golgi apparatus, where only the N-terminal transcription factor portion is cleaved, inducing the expression of ER stress response genes. (**B**) IRE1α/β exists as a monomer under normal conditions. When stimulated by unfolded proteins, it dimerizes and activates downstream signaling molecules TRAF2, NFκB, and JNK. Additionally, it induces the splicing of XBP1 mRNA, leading to the expression of XBP1s, which promotes the expression of ER stress response genes. (**C**) Similar to IRE1, PERK dimerizes and activates under ER stress. This activation leads to the phosphorylation of eIF2α and sequentially promotes the expression of ATF4. ATF4 then induces the expression of ER stress response genes. Abbreviations: ATF4, Activating Transcription Factor 4; ATF6α/β, Activating Transcription Factor 6 alpha/beta; BiP, Binding Immunoglobulin Protein; eIF2α, Eukaryotic Initiation Factor 2 alpha; ER, Endoplasmic Reticulum; IRE1α/β, Inositol-Requiring Enzyme 1 alpha/beta; JNK, c-Jun N-terminal Kinase; NFκB, Nuclear Factor kappa-light-chain-enhancer of activated B cells; PERK, Protein kinase RNA-like Endoplasmic Reticulum Kinase; S1P, Site-1 Protease; S2P, Site-2 Protease; TRAF2, TNF Receptor-Associated Factor 2; UPR, Unfolded Protein Response; XBP1, X-box Binding Protein 1; XBP1s, Spliced X-box Binding Protein 1.

In the Golgi, ATF6α/β undergoes cleavage by Site-1 and Site-2 proteases, releasing its N-terminal cytoplasmic domain [6,7]. This domain acts as a transcription factor, upregulating genes that enhance the protein-folding capacity of the ER and degrade misfolded proteins, thus mitigating ER stress. IRE1α/β normally exists as a monomer and dimerizes upon activation by unfolded proteins [8,9]. This dimerization recruits TRAF2, activating NFκB and JNK signaling pathways [10,11]. Additionally, IRE1α/β splices XBP1 mRNA to produce XBP1s, which acts as a transcription factor to upregulate ER stress response genes [12]. PERK also plays a significant role in the UPR by dimerizing upon ER stress activation. This dimerization phosphorylates eIF2α, leading to a reduction in general protein synthesis, which alleviates the burden on the ER [5,11]. In addition, it enhances the translation of ATF4, inducing the expression of genes involved in amino acid metabolism, redox reactions, and apoptosis, thereby contributing to cell survival under stress conditions [13] (Figure 1).

HEK293T cells are extensively used in the bioindustry for the production of recombinant proteins and viral vectors [14,15,16]. Their human origin makes them particularly suitable for producing biologics intended for human use, ensuring more accurate post-translational modifications and reducing immunogenicity compared to non-human cell lines like CHO cells [14]. These characteristics make HEK293T cells ideal for producing therapeutic proteins and viral vectors used in gene therapy, vaccine development, and cancer treatments [15,16,17]. However, despite these advantages, HEK293T cells may present limitations such as inconsistent protein expression and challenges in scalability for large-scale production. Among the proteins obtained from HEK293T cells, membrane proteins are of higher industrial value than cytosolic proteins due to their easier separation and purification process [18,19]. Given the high costs associated with purification, improving the productivity of membrane proteins derived from HEK293T cells is essential for their broader use in the industry [14,18,20]. Various strategies, including transient transfection, stable cell line development, and site-specific integration using recombinase-mediated cassette exchange, have been employed to enhance protein production in HEK293T cells [20,21,22,23,24]. Despite these advancements, challenges such as scalability, reproducibility, and the complexity of achieving stable high-yield production persist.

Since the UPR is an essential process for cell survival, particularly under stress conditions, numerous investigators revealed that this mechanism allows cells to minimize membrane protein production and other non-essential functions, thereby focusing resources on managing stress and promoting cell survival [1,11,25,26,27]. Instead of completely blocking the UPR, selective modulation of specific components, such as ATF6B, may help alleviate excessive stress responses and improve membrane protein production. In this study, the C-terminal domain of ATF6B—a regulatory element involved in controlling UPR intensity—was selectively deleted using CRISPR-Cas9 technology. This deletion was found to prevent overactivation of the UPR without inducing cytotoxicity, thereby establishing a cellular environment more favorable for membrane protein biosynthesis.

## 2. Materials and Methods

### 2.1. Cell Culture and Transfection

HEK293T cells were obtained from the Korean Cell Line Bank (Seoul, Republic of Korea), which also provided short tandem repeat (STR) profiling data to authenticate the cell line. The STR markers were as follows: D3S1358 (15,17), TH01 (7,9.3), D21S11 (28,30.2), Penta_E (7,15), D5S818 (8,9), D13S317 (12,14), D7S820 (11), D16S39 (9,13), CSF1PO (11,12), Penta_D (9,10), Amelogenin (X), vWA (16,19), D8S1179 (12,14), TPOX (11), FGA (23), D19S433 (18), and D2S1338 (19). The full STR profile is provided in Supplementary Table S1. Cells were maintained in Dulbecco’s Modified Eagle′s Medium (DMEM; Hyclone, Locan, UT, USA) supplemented with 10% fetal bovine serum (FBS; Hyclone, Logan, UT, USA), 50 units/mL penicillin, and 50 μg/mL streptomycin (GibcoBRL, Carlsbad, CA, USA) at 37 °C in humidified air containing 5% CO_2_. All plasmid DNA transfections in HEK293T cells were conducted using Lipofectamine 2000 (Thermo Fisher Scientific, Waltham, MA, USA), which was chosen for its high transfection efficiency and cell viability in HEK293T cells, as well as its compatibility with previously established protocols in our laboratory.

### 2.2. Construction of Plasmid DNA Vectors

**Figure 2:**
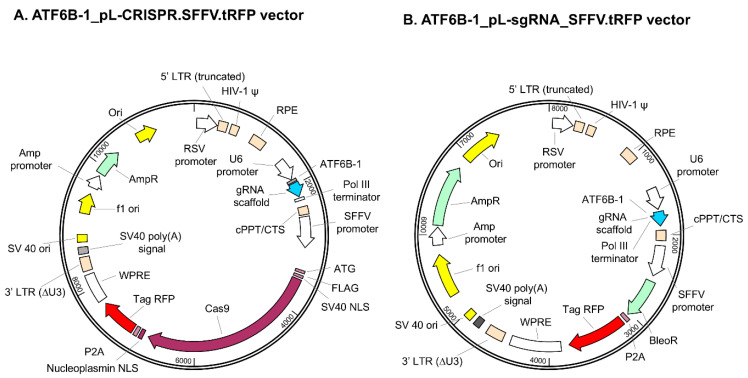
Schematic diagrams of lentiviral plasmids used for ATF6B-targeted CRISPR-Cas9 editing. (**A**) ATF6B-1_pL-CRISPR.SFFV.tRFP vector expressing Cas9 and sgRNA under the SFFV and U6 promoters, respectively. The construct includes TagRFP for visualization and nuclear localization signals (NLS). (**B**) ATF6B-1_pL-sgRNA_SFFV-ZeoR-tRFP vector expressing sgRNA with Zeocin resistance and TagRFP under the U6 and SFFV promoters, respectively.

The All-in-One vector system (named pL-CRISPR.EFS.tRFP), kindly provided by Benjamin Ebert (Addgene plasmid # 57819; http://n2t.net/addgene:57819; accessed on 21 June 2022; RRID:Addgene_57819), was utilized as the starting material (Table 1). To produce a stable cell line, a lentivirus vector of two vector systems was produced. First, to create a single guide RNA (sgRNA) expression vector named pL-sgRNA.EFS.Zeo.tRFP, the Cas9 gene was excised from the pL-CRISPR.EFS.tRFP vector, and a bleomycin resistance gene was inserted. Additionally, a lentivirus vector expressing Cas9 and neomycin resistance genes was synthesized using VectorBuilder (Guangzhou, China). A lentiviral vector facilitating the stable expression of a membrane-bound form of green fluorescent protein (mGFP) was engineered by cloning the GAP43 palmitoylation signal peptide sequence along with GFP into the pLVX-CMV-IRES-Puro vector (manufactured by Takara Bio, Inc., Otsu, Japan). The structural design and component organization of the lentiviral plasmids used in this study are illustrated in Figure 2A,B, which depict the Cas9/sgRNA expression vector and the sgRNA-only vector, respectively. These schematics are provided to enhance clarity regarding functional elements and selection markers.

This is a list of primers used to create CRISPR-Cas9 constructs for candidate genes. (For cloning, forward and reverse primers were annealed together, and then ligation was performed using T4 ligase).

### 2.3. Design and Construction of sgRNAs Targeting Candidate Genes

Utilizing the CRISPR RGEN Tool’s Cas-Designer (http://www.rgenome.net/cas-designer/, accessed on 21 June 2022), sgRNAs targeting ATF6A, ATF6B, IRE1A, IRE1B, and PERK were designed (one set of 3 sgRNAs for each target gene); ATF6A and ATF6B remove the C-terminal transmembrane domain (TMD), IRE1A and IRE1B suppress the expression of genes, and PERK removes the cytoplasmic domain (CPD) and protein kinase domain (PKD) at the C terminus. To create the designed sgRNA expression vector for genetic screening, oligonucleotide pairs encoding each sgRNA were synthesized through Bionics (Seoul, Republic of Korea). These oligonucleotide pairs were inserted into pL-CRISPR.EFS.tRFP to create a construct expressing each sgRNA. To produce stable cells, ATF6B-TMD-1 was inserted into the pL-sgRNA.EFS.Zeo.tRFP vector. The sgRNA targeting the transmembrane domain (TMD) of ATF6B was designed to induce deletion of the C-terminal region. Cells edited using this sgRNA are referred to as “ATF6B-ΔC” throughout this manuscript.

### 2.4. T7 Endonuclease I Assay (T7E1 Assay)

To evaluate the activity of each sgRNA, HEK293T cells were transfected with All-in-One vectors expressing each sgRNA. After 3 days of transfection, 5 × 10^6^ transfected cells were harvested, and the genomic DNA of each sample was purified using the G-DEX™ IIc Genomic DNA Extraction Kit (iNtRON Biotechnology, Seoul, Republic of Korea). Genomic DNA purification was performed according to the manufacturer’s instructions.

To obtain the amplified target gene PCR product for each sample, target regions were amplified through PCR (EzPCR™ HF 5x PCR Master Mix; Elpis Biotech, Daejeon, Republic of Korea) using the designed primer pair (Table 2). The first step of PCR conditions was 1 cycle of 5 min at 95 °C, followed by 10 cycles of 30 s at 95 °C, 30 s at 70 °C (annealing temperature was decreased by 1 °C per cycle to 60 °C), and 2 min at 72 °C. Next, the second step of PCR conditions was 30 cycles of 30 sec at 95 °C, 30 sec at 60 °C, and 2 min at 72 °C, finishing with 3 min incubation at 72 °C. After amplification of target genes, the hybrid DNA duplex is formulated by PCR cycling method as follows: 2 min at 95 °C, ramp down (−2 °C/s) at 95 °C to 85 °C, and (−0.1 °C/s) at 85 °C to 25 °C. For the T7E1 reaction, a 20 µL mixture (containing 2 µL of 10× NEB buffer-2, 10 units of T7E1 enzyme, and 10 µL of hybrid DNA duplex) was prepared, and the reaction was carried out at 37 °C for 20 min. T7E1 reactants were separated on a 1.5% agarose gel in TAE buffer containing 1× RedSafe Nucleic Acid Staining Solution (iNtRon Biotechnology) by electrophoresis and visualized under a Blue LED light.

This is a list of PCR primers used to analyze the results of candidate genes. These are T7E1 primers for PCR amplification of the target region of each candidate gene used in the T7E1 assay and primers for NGS for edited sequence analysis of ATF6B-TMD (ATF6B-ΔC), which is the final candidate.

### 2.5. Generation of Stable HEK293T Cell Lines with Cas9, mGFP, and Targeted sgRNAs

Lentiviral vectors were utilized to create HEK293T cell lines stably expressing Cas9 or mGFP. Lentiviruses containing either the pLV-Cas9.SFFV.Neo or the pLVX-CMV.mGFP.IRES.Puro vectors were transfected into HEK293T cells for this purpose. Selection of stable cell lines expressing Cas9 was achieved using G418 (1000 μg/mL; InvivoGen, San Diego, CA, USA), while those expressing mGFP were selected using Puromycin (2 μg/mL; InvivoGen) over a period of two weeks.

Lentiviral vectors were also utilized to create genome-edited cell lines to knock out the ATF6B gene by CRISPR-Cas9 technology. Briefly, HEK293T cells were transfected with lentiviruses carrying pL-sgRNA.EFS.Zeo.tRFP vectors, each containing a designed sgRNA targeting specific genes. Following the transduction process, the HEK293T cells infected with sgRNAs underwent a rigorous selection phase. This phase involved the application of Zeocin at a concentration of 200 μg/mL (InvivoGen) as the selective agent. This selection process was carried out over a period of two weeks, ensuring that only the cells successfully incorporating the sgRNA remained and proliferated.

### 2.6. Next-Generation Sequencing (NGS)

In total, 5 × 10^6^ cells were harvested, and the genomic DNA of each sample was purified. Genomic DNA purification was performed according to the manufacturer’s instructions. To obtain the amplified ATF6B gene PCR product for each sample, the ATF6B target region was amplified through PCR using the designed primer pair (Table 2). The first step of PCR conditions was 1 cycle of 5 min at 95 °C, followed by 10 cycles of 10 s at 95 °C, 10 s at 70 °C or 60 °C (annealing temperature was decreased by 1 °C per cycle), and 30 s at 72 °C. Next, the second step of PCR conditions was 30 cycles of 10 s at 95 °C, 10 s at 60 °C, and 30 s at 72 °C, finishing with 3 min incubation at 72 °C.

PCR products were separated on a 1.5% agarose gel in TAE buffer containing 1× RedSafe Nucleic Acid Staining Solution (iNtRon Biotechnology) by electrophoresis and visualized under a Blue LED light. After visualization of the PCR products, the gel was cut, and the PCR band that matched the expected size was collected. Collected PCR products were extracted using the Labopass GEL EXTRACTION KIT (Cosmogenetech, Seoul, Republic of Korea). Gel extraction was performed according to the manufacturer’s instructions. Extracted PCR products were analyzed through an NGS analysis company (Bionics, Seoul, Republic of Korea).

To assess potential off-target effects of the ATF6B sgRNA, in silico prediction was conducted using the Cas-OFFinder tool (http://www.rgenome.net/cas-offinder/, accessed on 4 February 2025) under mismatch conditions of 1–3 nucleotides. A total of 17 candidate off-target sites were identified. Genomic DNA was extracted, and PCR amplification was performed to generate 900–1200 bp products spanning each predicted site. The amplified products were subjected to next-generation sequencing (BitSeq, Bionics, Seoul, Republic of Korea). The resulting sequence data were analyzed using the Cas-Analyzer tool (http://www.rgenome.net/cas-analyzer/, accessed on 3 April 2025) to detect indel events. No insertions or deletions were observed at any of the predicted off-target sites.

### 2.7. Determination of Membranous Protein Yields

For the extraction of membrane proteins, 5 × 10^6^ cells from both ATF6B-ΔC and wild-type HEK293T cell lines were collected. The membrane protein extraction was performed using Mem-PER Plus Membrane Protein Extraction Kit (Thermo Fisher, Inc., Waltham, MA, USA) according to the manufacturer’s instructions. The harvested cells were washed 2 times with Cell Wash Solution (info) and permeabilized with 0.75 mL of Permeabilization Buffer (info) (incubation at 4 °C for 10 min with mixing). After incubation, the samples were centrifuged for 15 min (16,000× *g* at 4 °C) to separate the supernatant and pellet. The supernatant was discarded, and the pellet was resuspended in 0.5 mL of Solubilization Buffer (info). Then, the resuspended samples were incubated for 30 min at 4 °C with mixing. After that, the mixed samples were separated into supernatant and pellet through centrifugation (15 min/16,000× *g*/4 °C). Finally, the membrane protein samples (supernatant) were obtained from the centrifugates. The Bradford assay was employed to quantify the production yield of membrane proteins. A total of 20 µL of the extracted membrane proteins was diluted by 180 µL of D.W, and 10 µL of the diluted samples were mixed with 200 µL of 1× Bradford reagent (Bio-Rad Protein Assay Dye Reagent Concentrate; Bio-Rad, Hercules, CA, USA). After 5 min incubation, the absorbance was measured at 595 nm using a photometer (SpectraMax^®^ ABS; Molecular Devices, San Jose, CA, USA).

### 2.8. Flow Cytometric Analysis

To measure the mGFP intensity of ATF6B-ΔC mGFP-stable HEK293T cells, ATF6B-ΔC and WT mGFP-stable HEK293T cells were seeded into 100 mm cell culture dish prior to the experiment. Cells were cultured to approximately 100% confluency and then harvested by trypsinization. Harvested cells were washed twice with PBS and subsequently resuspended in PBS. Resuspended cells were transferred to new FACS tubes, and GFP intensity was measured through an Attune NxT flow cytometer (Thermo Fisher, Inc.).

### 2.9. Quantitative Real-Time PCR for UPR-Related Gene Expression

To evaluate the impact of ATF6B deletion on other UPR pathway components, total RNA was extracted from wild-type and ATF6B-ΔC HEK293T cells using TRIzol reagent (Invitrogen, Waltham, MA, USA) according to the manufacturer’s instructions. cDNA was synthesized using the RevertAid First Strand cDNA Synthesis Kit (Thermo Fisher Scientific). Quantitative real-time PCR was performed using SYBR Green PCR Master Mix (Applied Biosystems, Waltham, MA, USA) on a StepOnePlus™ Real-Time PCR System (Applied Biosystems). The relative expression levels of IRE1α, IRE1β, PERK-CPD, and PERK-PKD were determined using the ΔΔCt method, with GAPDH as the internal control. The following primers were used: IRE1α (forward: 5′-AGGAGGCTGAGGAAGGAGAA-3′, reverse: 5′-TGGTAGGAGGCAGTGAGGAT-3′), IRE1β (forward: 5′-ACGAGGAGAAGGAGGAGATG-3′, reverse: 5′-CTGTGGCAGTAGTAGGAGGA-3′), PERK-CPD (forward: 5′-GGAAGTGTTCCAGCTTCAGG-3′, reverse: 5′-GTCATCAGGCAGCTTTCTCA-3′), PERK-PKD (forward: 5′-AGATGTGGCTGGTGATGTTG-3′, reverse: 5′-TGGCTGAAGATGAGGTTGAA-3′), and GAPDH (forward: 5′-GAAGGTGAAGGTCGGAGT-3′, reverse: 5′-GAAGATGGTGATGGGATTTC-3′).

### 2.10. Statistical Analysis

The data are expressed as means ± SEM. An unpaired *t*-test was utilized to compare two groups, considering *p* < 0.05 as the threshold for statistical significance. The analyses were derived from three independent experimental replicates.

## 3. Results

### 3.1. Enhancing sgRNA Efficiency for ATF6B Gene Editing

**Figure 3:**
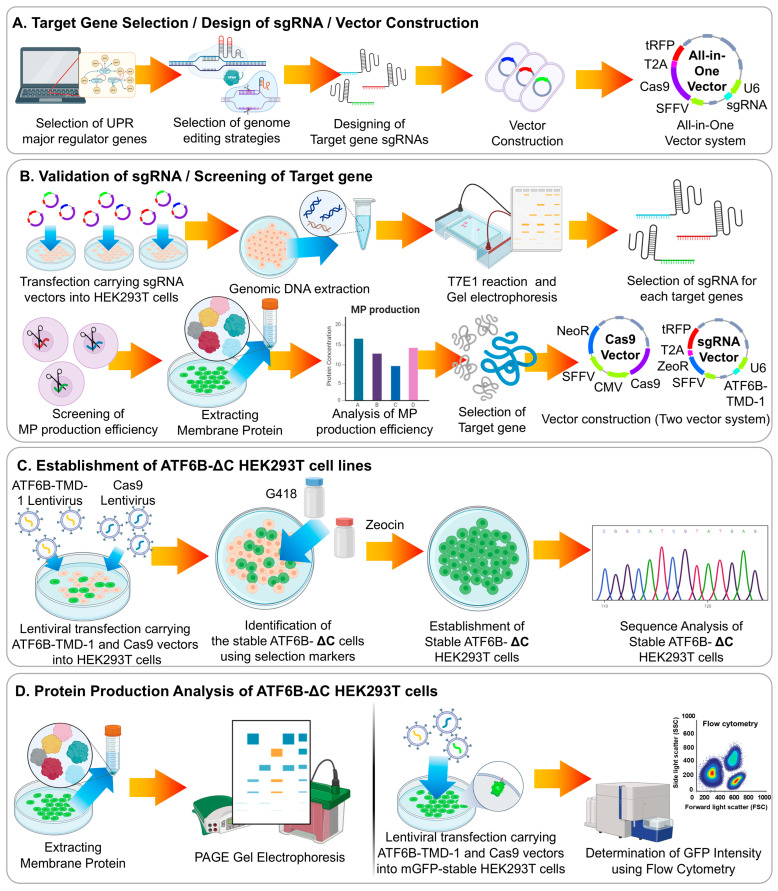
Workflow for developing a HEK293T cell line with enhanced membrane protein production through ATF6B gene editing. (**A**) The literature review and sgRNA design. Candidate genes were identified through a comprehensive literature review. sgRNAs targeting these genes were designed and incorporated into an All-in-One vector system, which expresses both Cas9 and sgRNA in a single vector. (**B**) sgRNA efficiency assessment. The constructed sgRNAs were introduced into HEK293T cells. The efficiency of each sgRNA was assessed using the T7E1 assay, which detects mismatched DNA by cleaving it with T7 Endonuclease I. The sgRNAs with the most significant cleaved band patterns were selected for further experiments. (**C**) Lentiviral packaging and infection. The selected sgRNA and Cas9 were packaged into lentivirus particles and used to infect HEK293T cells. Stable expression was ensured by selecting cells with Zeocin for sgRNA and G418 for Cas9. Sequencing was then performed to confirm the genetic editing patterns in the selected cells. (**D**) Membrane protein production analysis. Membrane proteins were purified from both the stable cell line and wild-type (WT) cell line to compare production levels. The effect of ATF6B editing on membrane protein production was indirectly assessed using flow cytometry by analyzing cells expressing Membrane-GFP.

In this study, the experimental steps were carried out in the following manner (Figure 3). First, a literature review identified candidate genes, and sgRNAs targeting these genes were designed. Vectors were constructed using an All-in-One system, where both Cas9 and sgRNA are expressed in a single vector. The sgRNAs were then introduced into HEK293T cells, and their efficiency was assessed using the T7E1 assay to select the most efficient sgRNAs. Next, the selected sgRNA and Cas9 were packaged into lentivirus particles and used to infect HEK293T cells. Stable expression was ensured by selecting cells with Zeocin for sgRNA and G418 for Cas9. Sequencing confirmed the genetic editing patterns in the selected cells. Finally, membrane proteins were purified from both the stable cell line and wild-type (WT) cell line to compare production levels. Membrane protein production in ATF6B-edited cells was indirectly assessed using flow cytometry based on GFP intensity in cells expressing membrane-anchored GFP.

**Figure 4:**
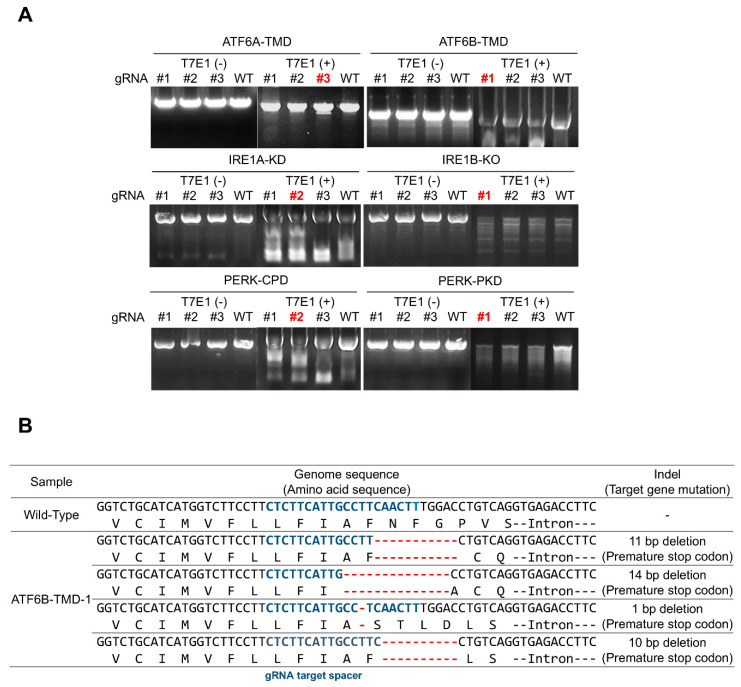
Screening and evaluation of sgRNA efficiency targeting multiple UPR-related genes and detailed mutation analysis of ATF6B. (**A**) T7E1 assay results for sgRNAs designed to target several UPR-related genes, including ATF6A-TMD, ATF6B-TMD (ATF6B-ΔC), IRE1A-KD, IRE1B-KO, PERK-CPD, and PERK-PKD. HEK293T cells were transfected with each sgRNA construct, and genomic DNA was extracted for mismatch detection using the T7E1 assay. Among the candidates, ATF6B-TMD displayed the strongest cleaved band intensity, indicating the highest editing efficiency. Candidate gDNA sequences are indicated in red. (**B**) To further characterize the editing patterns of ATF6B, sequencing of the target region was conducted using Cas-Analyzer software (Cas-Database the information is updated based on Ensembl 86). The blue-colored sequence indicates the sgRNA recognition site. In ATF6B-ΔC edited cells, predominant deletions of 11, 14, 1, and 10 base pairs were observed, which disrupted exon integrity, introduced frameshifts, and generated premature stop codons, ultimately suppressing ATF6B expression.

In the first step of the experiment, sgRNAs targeting ATF6A-TMD (transmembrane domain), ATF6B-ΔC, IRE1A-KD (knockdown), IRE1B-KO (knockout), PERK-CPD (cytoplasmic domain), and PERK-PKD (protein kinase domain) were introduced into HEK293T cells. Their efficiency was assessed using the T7E1 assay, which detects mismatched DNA by cleaving it with T7 endonuclease I. Among these, ATF6B-ΔC showed the most significant results with the highest cleaved band patterns, indicating it was the most efficient sgRNA. A representative gel image from the T7E1 assay showing cleavage bands for each sgRNA, including ATF6B-TMD#1, is presented in Figure 4A. To further examine the gene editing patterns, sequencing was conducted (Figure 4B), which identified predominant 11, 14, 1, and 10 bp nucleotide deletions in ATF6B-ΔC edited cells. Comparative analysis with the gene sequence showed that the 11 and 14 bp deletions disrupted exon sequences, causing exon skipping and subsequent suppression of normal protein expression. Additionally, the 1 bp deletion introduced a premature stop codon, and the 10 bp deletion caused a frameshift mutation, both further inhibiting protein expression.

### 3.2. Establishment of ATF6B-ΔC HEK293T Cells Using CRISPR-Cas9 Technology

**Figure 5:**
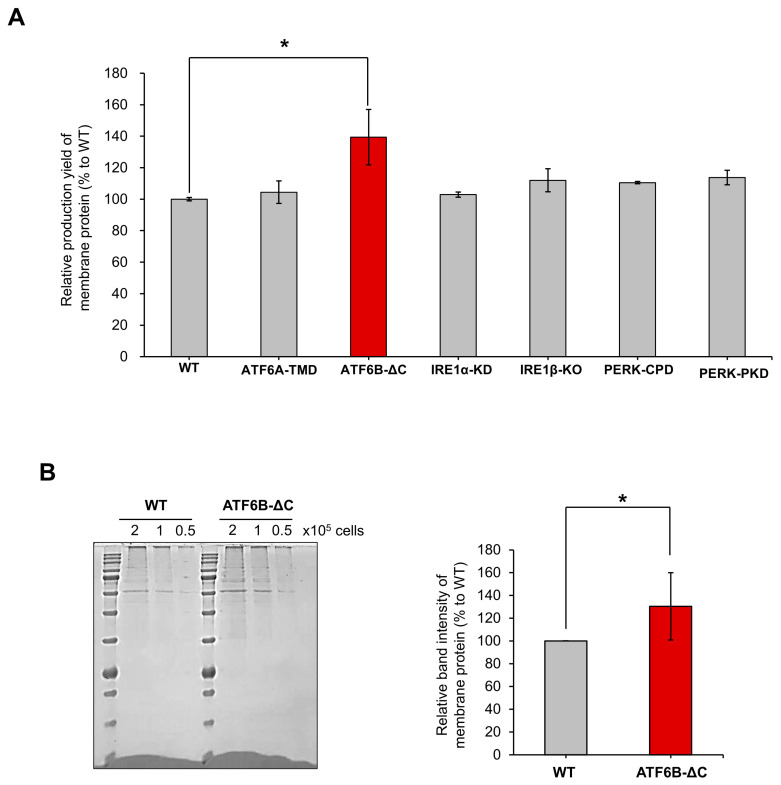
Comparison of membrane protein production between wild-type (WT) and ATF6B-ΔC HEK293T cells. (**A**) Relative production yield of membrane proteins in HEK293T cells after gene editing. The graph shows that ATF6B-ΔC cells exhibited a significant increase in membrane protein production compared to WT and other gene-edited cells (ATF6A-TMD, IRE1A-KD, IRE1B-KO, PERK-CPD, PERK-PKD). (**B**) Quantitative comparison of membrane proteins. Purified proteins from WT, ATF6B-ΔC, and other gene-edited cells were separated on a PAGE gel at three different cell concentrations (2, 1, and 0.5 × 10^5^ cells). The ATF6B-ΔC cell line displayed more intense bands than the WT and other gene-edited cells, indicating higher protein production. The bar graph shows the relative band intensity of membrane proteins, demonstrating an increase in the ATF6B-ΔC cells compared to WT and other gene-edited cells. Data are presented as means ± SEM from three independent experiments, with a significant difference marked by * *p* < 0.05.

HEK293T cells were transfected with lentiviral vectors containing Cas9 and sgRNAs targeting ATF6A-TMD, ATF6B-ΔC, IRE1A-KD, IRE1B-KO, PERK-CPD, and PERK-PKD, respectively. Successful transfection of HEK293T cells was confirmed using G418 and Zeocin as selection markers. The membrane protein production levels of these gene-edited cells were compared with those of wild-type (WT) HEK293T cells. The production of membrane proteins purified from the ATF6B-ΔC HEK293T cells showed a significant increase of approximately 40 ± 17.6% compared to the WT cells (Figure 5A). This increase was not observed in cells with IRE1 or PERK disruption, indicating that the enhancement is specific to ATF6B deletion rather than a result of generalized protein synthesis.

For quantitative comparison of the proteins, the purified proteins were separated on a PAGE gel at different cell concentrations. When an equal number of cells were loaded, the ATF6B-ΔC cell line displayed more intense bands than the WT and other gene knockout cells, indicating higher protein production. Quantitative analysis of the band intensity revealed an increase in membrane protein production in the ATF6B-ΔC cells (Figure 5B).

### 3.3. Assessment of Enhanced Membrane Protein Production in HEK293T Cells with C-Terminally Deleted ATF6B (ATF6B-ΔC)

**Figure 6:**
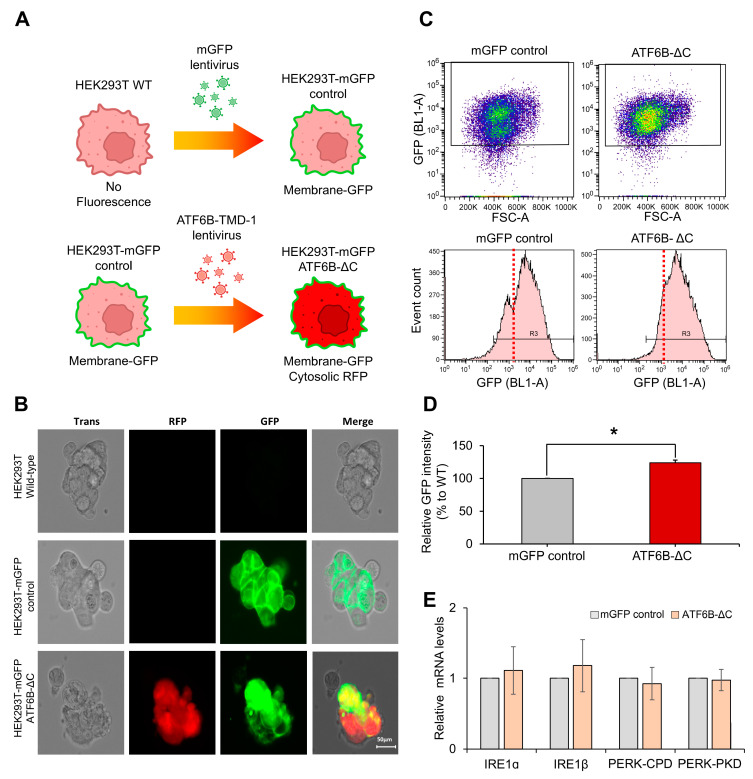
Comparison of membrane protein productivity in live HEK293T-ATF6B-ΔC cells. (**A**) Schematic illustration of target gene expression. Membrane-GFP genes were constructed and introduced into HEK293T cells to create a stable mGFP-HEK293T cell line. This stable cell line enabled the visualization of membrane-GFP expression through fluorescence microscopy. Additionally, a lentiviral vector containing ATF6B-ΔC gRNA and RFP was delivered into these cells. The successful delivery and expression of this vector were indirectly confirmed by the presence of cytosolic RFP, serving as a visual marker for the cells expressing the ATF6B-ΔC construct. (**B**) Representative fluorescence microscopic images showing the distinct expression patterns of the cell lines. HEK293T WT cells displayed no fluorescence, confirming the absence of GFP and RFP signals. HEK293T-mGFP cells exhibited GFP expression, visible in the GFP channel, and HEK293T-mGFP-ATF6B-ΔC cells showed fluorescence in both GFP and RFP channels, confirming the successful integration and expression of both GFP and RFP in the edited cell line. (**C**) Flow cytometry showing the changes in GFP intensity in HEK293T-mGFP control and HEK293T-mGFP-ATF6B-ΔC cells. Analysis was conducted using dot plots (top) and histograms (bottom). Two groups were confirmed for HEK293T-mGFP control: a low GFP intensity group (10^3^) and a high GFP intensity group (10^4^), but ATF6B-ΔC cells were mostly in the high GFP intensity group (10^4^). (**D**) Median GFP intensity comparison. The difference in GFP intensity between control cells and HEK293T cells with C-terminally deleted ATF6B (ATF6B-ΔC cells) was compared and analyzed through the median value of GFP intensity. A 23.9 ± 4.2% increase in GFP intensity was confirmed in ATF6B-ΔC cells compared to WT cells. (**E**) Expression of IRE1 and PERK pathway components in ATF6B-ΔC and wild-type HEK293T cells. Relative mRNA levels of IRE1α, IRE1β, PERK-CPD, and PERK-PKD were quantified by qRT-PCR. No significant differences were observed between WT and ATF6B-ΔC cells. Data represent mean ± SEM from three independent experiments. Values are presented as mean ± standard deviation from three independent experiments. * *p* < 0.05.

To confirm the increase in membrane protein in live cells, Membrane-GFP genes were constructed and introduced into HEK293T cells to create a stable mGFP-HEK293T cell line (Figure 6A). Additionally, a lentiviral vector containing ATF6B-ΔC gRNA and RFP was delivered into these cells, and successful transduction was indirectly confirmed by the presence of cytosolic RFP.

Fluorescence microscopic imaging revealed distinct expression patterns among the cell lines (Figure 6B). HEK293T wild-type cells displayed no fluorescence, confirming the absence of both GFP and RFP signals. In contrast, HEK293T-mGFP cells exhibited clear GFP fluorescence, while HEK293T-mGFP-ATF6B-ΔC cells showed both GFP and RFP signals, indicating successful co-expression. Notably, GFP fluorescence corresponding to membrane protein expression was uniformly localized along the cell membrane in both wild-type and ATF6B-ΔC cells, indicating no detectable differences in subcellular distribution between the two groups.

Flow cytometry was used to quantify GFP intensity after maintaining the stable cell lines for more than two weeks (Figure 6C). To ensure accurate comparison across samples, fluorescence analysis was performed using the same number of cells in each group during flow cytometric measurement. The flow cytometric data showed that wild-type cells consisted of two distinct populations—low GFP intensity (10³) and high GFP intensity (10⁴). However, ATF6B-ΔC cells were predominantly within the high GFP intensity population. A comparative analysis revealed that ATF6B-ΔC cells exhibited a 23.9 ± 4.2% increase in median GFP intensity compared to wild-type cells (Figure 6D). This enhancement was consistently observed across more than three independent experiments, confirming the reproducibility of the result.

To confirm the specificity of the CRISPR-Cas9 editing and rule out potential off-target effects, we performed both bioinformatic prediction and experimental validation. Using the Cas-OFFinder tool, 17 potential off-target sites were identified under mismatch conditions of 1–3 base pairs (Supplementary Table S2). Each site was PCR-amplified using specific primers (Supplementary Table S3), and the resulting ~900–1200 bp products were subjected to next-generation sequencing (BitSeq). Indel analysis was conducted using Cas-Analyzer, and no insertions or deletions were detected at any of the predicted sites (Supplementary Table S4), indicating that the sgRNA used for ATF6B targeting exhibited high specificity under the experimental conditions.

To further assess the broader effects of ATF6B deletion on the unfolded protein response (UPR), the expression levels of other key UPR components—IRE1α, IRE1β, PERK-CPD, and PERK-PKD—were examined in ATF6B-ΔC and wild-type HEK293T cells. As shown in Figure 6E, no significant changes in mRNA levels were observed between the two groups. These findings indicate that the enhanced membrane protein production in ATF6B-ΔC cells is not accompanied by major transcriptional alterations in other UPR pathways, supporting the specificity of the ATF6B-targeted modulation.

## 4. Discussion

This study aimed to enhance membrane protein production in HEK293T cells by using CRISPR-Cas9 technology to edit the ATF6B gene. The experimental approach involved optimizing sgRNA efficiency, validating gene editing, and comparing membrane protein production levels between edited and wild-type cells. The sgRNAs targeting ATF6A-TMD, ATF6B-ΔC, IRE1A-KD, IRE1B-KO, PERK-CPD, and PERK-PKD were designed, with ATF6B-ΔC showing the highest efficiency in the T7E1 assay. Sequencing of the ATF6B-ΔC edited cells revealed predominant 11, 14, 1, and 10 bp deletions, which disrupted exon sequences, caused exon skipping, and introduced premature stop codons, leading to suppression of normal protein expression. Comparative analysis of membrane protein production showed a significant increase of approximately 40 ± 17.6% in ATF6B-ΔC cells compared to wild-type cells. Quantitative analysis using PAGE gel and flow cytometry confirmed the enhanced production, with a 23.9 ± 4.2% increase in GFP intensity in ATF6B-ΔC cells.

To investigate the potential off-target effects of ATF6B sgRNA, we used the Cas-OFFinder tool (http://www.rgenome.net/cas-offinder/) to identify 17 candidate sites under 1–3 bp mismatch conditions. PCR amplicons (900–1200 bp) covering these regions were subjected to NGS (BitSeq, Bionics; Seoul, Republic of Korea), and indel analysis was performed using Cas-Analyzer. No indel mutations were detected at any of the 17 predicted sites, suggesting high specificity of the sgRNA used. A detailed list of the predicted off-target sites and their sequencing results is presented in Supplementary Table S3. These results indicate that the deletion of ATF6B’s C-terminal domain significantly enhances membrane protein productivity by upregulating the UPR pathway and increasing the cell’s capacity to handle misfolded proteins. This novel approach could have significant implications for the biotechnology and pharmaceutical industries, where membrane proteins are critical targets for drug development and other applications.

Despite the technical challenges associated with membrane protein purification, even moderate improvements in yield can have a profound industrial impact. Membrane proteins are inherently difficult to purify and stabilize due to their hydrophobic nature and structural complexity. However, when such proteins are used as raw materials for biopharmaceutical development, an increase of approximately 20% in production—as demonstrated in this study—represents more than a simple numerical improvement. It directly contributes to lowering manufacturing costs, improving production efficiency, and enhancing scalability. These benefits are particularly critical in the development of high-value or rare membrane protein-based therapeutics, where production bottlenecks often limit progress. Enhancing yield in this context strengthens commercial viability and supports broader accessibility to innovative therapies.

**Figure 7:**
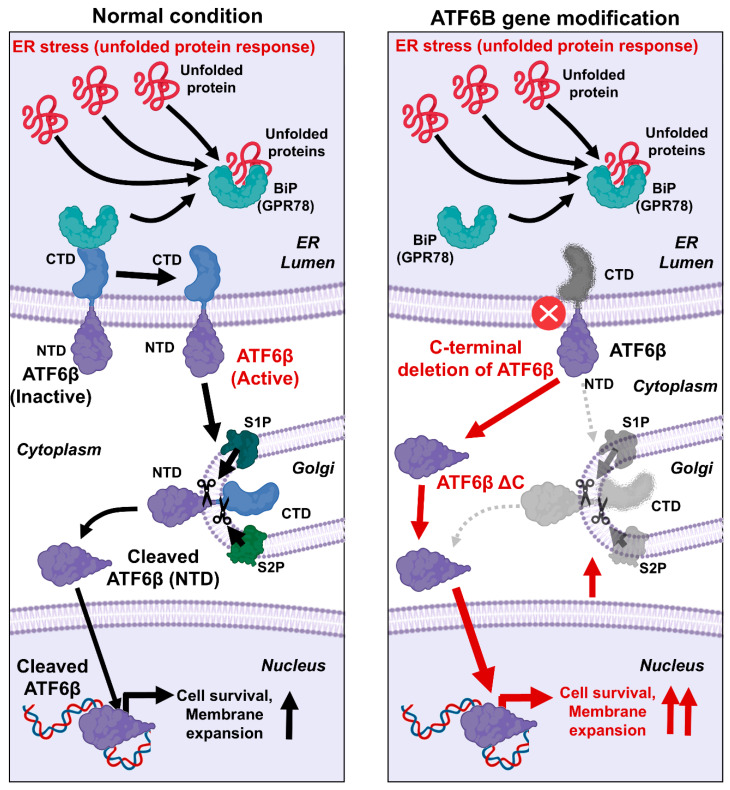
Possible mechanism by which C-terminal domain deletion of ATF6B induces enhanced membrane protein productivity. In the UPR process, BiP (GPR78) senses ER stress and normally binds to ATF6B to keep it inactive. However, when BiP binds to unfolded or misfolded proteins during ER stress, it separates from ATF6B. This separation allows ATF6B to move to the Golgi complex, where it undergoes proteolytic cleavage. The cleavage releases the N-terminal cytoplasmic domain of ATF6B, which then translocates to the nucleus and functions directly as a transcription factor. This transcription factor upregulates UPR target genes that are involved in enhancing the production of molecular chaperones and components of the ER-associated degradation pathway, thereby mitigating ER stress. In this study, targeting the C-terminal domain of ATF6B using CRISPR-Cas9 technology led to the deletion of this region, resulting in the expression of its cytoplasmic domain and activation of its transcriptional activity. This modification significantly enhanced membrane protein production by increasing the cell’s capacity to manage and process misfolded proteins. Abbreviations: ATF6, Activating Transcription Factor 6; BiP (Binding Immunoglobulin Protein, also known as GRP78 (Glucose-Regulated Protein 78); CTD, C-terminal domain; NTD, N-terminal domain.

The possible mechanism by which the C-terminal deletion of ATF6B enhances membrane protein productivity is as follows (Figure 7). The UPR is a crucial cellular process that mitigates the effects of ER stress by enhancing the production of molecular chaperones, degrading misfolded proteins, and attenuating protein synthesis [1,2,3]. The UPR is primarily regulated by three ER membrane-associated proteins: ATF6, PERK, and IRE1. ATF6, upon sensing ER stress, is transported to the Golgi apparatus, where it undergoes proteolytic cleavage by the Site-1 and Site-2 proteases (S1P and S2P) [1,3]. This cleavage releases the N-terminal cytoplasmic domain of ATF6, which then translocates to the nucleus to function as a transcription factor. This domain upregulates UPR target genes, including those encoding chaperones and components of the ER-associated degradation pathway. In this research, the C-terminal domain of ATF6B was specifically targeted using CRISPR-Cas9 technology. By deleting this region, it was hypothesized that the N-terminal cytoplasmic domain of ATF6B would be more readily available to enter the nucleus and exert its transcriptional effects. The results confirmed our hypothesis, showing a significant increase in membrane protein production in the ATF6B-ΔC cells compared to wild-type cells. This enhancement can be attributed to the intensified UPR, which maintains ER homeostasis and increases the cell’s capacity to handle misfolded proteins, thereby facilitating greater membrane protein synthesis [25,26]. The successful modification of ATF6B-ΔC led to the upregulation of UPR target genes, thereby enhancing the ER’s protein folding and processing capacity. This not only underscores the importance of ATF6B in the UPR pathway but also demonstrates a novel approach to augmenting membrane protein production in HEK293T cells. The successful deletion of the ATF6B C-terminal domain resulted in the upregulation of UPR target genes, enhancing the ER’s protein folding and processing capacity. This finding highlights the critical role of ATF6B in the UPR pathway and presents a novel strategy to boost membrane protein production in HEK293T cells.

The specific deletions in the ATF6B gene, including 11, 14, 1, and 10 bp deletions, were observed in this study as a result of the precise cutting action of the CRISPR-Cas9 system. These deletions led to the disruption of exon sequences, causing exon skipping and the introduction of premature stop codons. The CRISPR-Cas9 system was designed with sgRNAs targeting specific regions of the ATF6B gene, which directed the Cas9 nuclease to create double-strand breaks at these sites. During the repair process, the non-homologous end joining (NHEJ) pathway, which often results in insertions or deletions, repaired the breaks inaccurately, leading to the observed deletions. These genetic alterations disrupted the normal reading frame of the ATF6B gene, producing truncated, non-functional proteins or leading to the complete absence of ATF6B protein due to nonsense-mediated decay. Consequently, the disruption of the ATF6B gene by these deletions could lead to enhanced upregulation of UPR target genes and improved ER protein folding and processing capacity. This result highlights the critical role of ATF6B in the UPR pathway and presents a novel strategy to boost membrane protein production in HEK293T cells. Notably, despite the loss of ATF6B function, no significant reduction in cell viability was observed, suggesting that compensatory ER stress pathways may have helped preserve cell survival.

While the enhancement of membrane protein production via ATF6B editing holds significant promise for drug discovery and biomanufacturing, translating this approach to industrial-scale applications presents several challenges. One major concern is the long-term stability of the edited cell line. Gene editing, particularly when targeting stress response pathways like ATF6, may lead to subtle changes in cell growth behavior or genomic stability over extended culture periods, which could reduce productivity over time. Moreover, applying this system in large-scale bioreactor environments requires careful optimization, as cellular responses may vary under different culture conditions. These aspects highlight the need for further development before this platform can be widely adopted in industrial bioprocesses.

This study has several limitations. Although the C-terminal deletion of ATF6B significantly improved membrane protein production, the long-term stability and viability of the edited cells under large-scale production conditions remain untested. Potential off-target effects of CRISPR-Cas9 editing were not extensively evaluated, and the study focused solely on ATF6B, leaving other potentially synergistic genes unexplored. Additionally, the use of GFP as a reporter limits generalizability to other membrane proteins. Despite these limitations, this study offers a valuable proof-of-concept for using CRISPR-Cas9 to enhance membrane protein production and presents a novel strategy for modulating the UPR pathway to improve bioproduction efficiency.

In a broader context, the development of gene-edited cell lines for enhanced protein production also aligns with global efforts to address sustainability and food security challenges. As highlighted by Saleem et al. [28], biotechnology plays an essential role in achieving the Sustainable Development Goals (SDGs) by improving resource efficiency and supporting resilient systems under climate pressure. Our approach, while focused on biopharmaceutical production, may contribute to these broader goals by promoting more efficient and scalable biologics manufacturing platforms. In summary, this study demonstrates that CRISPR-Cas9-mediated deletion of the ATF6B transmembrane domain significantly enhances membrane protein production in HEK293T cells. Optimizing sgRNA efficiency and validating gene editing revealed that ATF6B-ΔC cells exhibited deletions that disrupted exon sequences and introduced premature stop codons, effectively suppressing normal protein expression. The gene editing resulted in a substantial increase in membrane protein production, highlighting the potential of targeting the ATF6B gene to upregulate the UPR pathway and improve cellular capacity for managing misfolded proteins. This approach holds promise for advancing biotechnology and pharmaceutical applications where efficient membrane protein production is critical.

## Figures and Tables

**Table 1:** Vector construction primers used in the study.

Primer Name	Description	Primer Sequences
ATF6A-TMD-1_F	Primer for CRISPR-Cas9 construction (ATF6A-TMD-1)	CACCGTCATTTCCTTTAGAACCAG
ATF6A-TMD-1_R		AAACCTGGTTCTAAAGGAAATGAC
ATF6A-TMD-2_F	Primer for CRISPR-Cas9 construction (ATF6A-TMD-2)	CACCGCAACTCTTCGCTTTGGACT
ATF6A-TMD-2_R		AAACAGTCCAAAGCGAAGAGTTGC
ATF6A-TMD-3_F	Primer for CRISPR-Cas9 construction (ATF6A-TMD-3)	CACCGACTGAACTATGGACCTATG
ATF6A-TMD-3_R		AAACCATAGGTCCATAGTTCAGTC
ATF6B-TMD-1_F	Primer for CRISPR-Cas9 construction (ATF6B-TMD-1)	CACCGTCTTCATTGCCTTCAACTT
ATF6B-TMD-1_R		AAACAAGTTGAAGGCAATGAAGAC
ATF6B-TMD-2_F	Primer for CRISPR-Cas9 construction (ATF6B-TMD-2)	CACCGCTTCAACTTTGGACCTGTC
ATF6B-TMD-2_R		AAACGACAGGTCCAAAGTTGAAGC
ATF6B-TMD-3_F	Primer for CRISPR-Cas9 construction (ATF6B-TMD-3)	CACCGAAGGAAGGTCTCACCTGAC
ATF6B-TMD-3_R		AAACGTCAGGTGAGACCTTCCTTC
IRE1A-KD-1_F	Primer for CRISPR-Cas9 construction (IRE1A-KD-1)	CACCGCTTATTTCTCATGGCTCGG
IRE1A-KD-1_R		AAACCCGAGCCATGAGAAATAAGC
IRE1A-KD-2_F	Primer for CRISPR-Cas9 construction (IRE1A-KD-2)	CACCGCAGTTTGTAAATGCTGGGG
IRE1A-KD-2_R		AAACCCCCAGCATTTACAAACTGC
IRE1A-KD-3_F	Primer for CRISPR-Cas9 construction (IRE1A-KD-3)	CACCGCTGCTTACCTTATTTCTCA
IRE1A-KD-3_R		AAACTGAGAAATAAGGTAAGCAGC
IRE1B-KO-1_F	Primer for CRISPR-Cas9 construction (IRE1B-KO-1)	CACCGCCAGGCGCTATGGCGAGTG
IRE1B-KO-1_R		AAACCACTCGCCATAGCGCCTGGC
IRE1B-KO-2_F	Primer for CRISPR-Cas9 construction (IRE1B-KO-2)	CACCGCGCACTCGCCATAGCGCCT
IRE1B-KO-2_R		AAACAGGCGCTATGGCGAGTGCGC
IRE1B-KO-3_F	Primer for CRISPR-Cas9 construction (IRE1B-KO-3)	CACCGCCGCACTCGCCATAGCGCC
IRE1B-KO-3_R		AAACGGCGCTATGGCGAGTGCGGC
PERK-CPD-1_F	Primer for CRISPR-Cas9 construction (PERK-CPD-1)	CACCGAACAACGTTTATTGTGCGC
PERK-CPD-1_R		AAACGCGCACAATAAACGTTGTTC
PERK-CPD-2_F	Primer for CRISPR-Cas9 construction (PERK-CPD-2)	CACCGTTCCATCCTCATCCTCACA
PERK-CPD-2_R		AAACTGTGAGGATGAGGATGGAAC
PERK-CPD-3_F	Primer for CRISPR-Cas9 construction (PERK-CPD-3)	CACCGTACCCTGTGAGGATGAGGA
PERK-CPD-3_R		AAACTCCTCATCCTCACAGGGTAC
PERK-PKD-1_F	Primer for CRISPR-Cas9 construction (PERK-PKD-1)	CACCGTTGAGCCAATTCAATGCCT
PERK-PKD-1_R		AAACAGGCATTGAATTGGCTCAAC
PERK-PKD-2_F	Primer for CRISPR-Cas9 construction (PERK-PKD-2)	CACCGCACGTCCCAGGCATTGAAT
PERK-PKD-2_R		AAACATTCAATGCCTGGGACGTGC
PERK-PKD-3_F	Primer for CRISPR-Cas9 construction (PERK-PKD-3)	CACCGTTCAATGCCTGGGACGTGG
PERK-PKD-3_R		AAACCCACGTCCCAGGCATTGAAC

**Table 2:** PCR primers used in this study.

Primer Name	Description	Primer Sequences
ATF6A-T7E1_F	Target gene amplification for T7 endonuclease I (T7E1) assay (Product: 1191 bp)	TCCCTGCTGGAAAAGGGAACC
ATF6A-T7E1_R		TGACAGCACCACCATTCGGTC
ATF6B-T7E1_F	Target gene amplification for T7 endonuclease I (T7E1) assay (Product: 1252 bp)	TGGCTGACAACCAGCAGCTC
ATF6B-T7E1_R		AGCTTTGGGTCCCCCTCACT
ATF6B-NGS_F	Target gene amplification for NGS (Product: 344 bp)	TGGAGGCCCTGCTGGCTGAAGTAA
ATF6B-NGS_R		TGGCCAGACACATCATCGGTGCCA
IRE1A-T7E1_F	Target gene amplification for T7 endonuclease I (T7E1) assay (Product: 1401 bp)	GGGCAGGTTGCAAAAGTCGC
IRE1A-T7E1_R		TGACCAGGCCCTCAGTCACA
IRE1B-T7E1_F	Target gene amplification for T7 endonuclease I (T7E1) assay (Product: 1812 bp)	TGGCGTGGGGGATAAGGTCT
IRE1B-T7E1_R		TACTCCGTTGGGGCCGAGTT
PERK-CPD-T7E1_F	Target gene amplification for T7 endonuclease I (T7E1) assay (Product: 1223 bp)	GAGAGGAACAAACGAAGCACAC
PERK-CPD-T7E1_R		ACCAGGCTAAGCGAGAGCAC
PERK-PKD-T7E1_F	Target gene amplification for T7 endonuclease I (T7E1) assay (Product: 1254 bp)	TGTCAGGTAGTGCCCACCCA
PERK-PKD-T7E1_R		ACAGCTGGTTAATTGGCAGCAC

## Data Availability

The datasets generated and/or analyzed during the current study are available from the corresponding author upon reasonable request.

## Supplementary Materials

The following supporting information can be downloaded at: https://www.mdpi.com/article/10.3390/bioengineering12040409/s1, Table S1: STR profiling of HEK298T cells; Table S2: Prediction of on/off target; Table S3: Primer for Off-target analysis; Table S4: Off-target analysis of HEK293T cell resulting from ATF6B targeting.
