# Supplementary figures and images for: CRISPR-Cas9-Mediated ATF6B Gene Editing Enhances Membrane Protein Production in HEK293T Cells
Source: Bioengineering (Basel). 2025 Apr 11;12(4):409. doi: 10.3390/bioengineering12040409 (PMC12025008; doi:10.3390/bioengineering12040409)

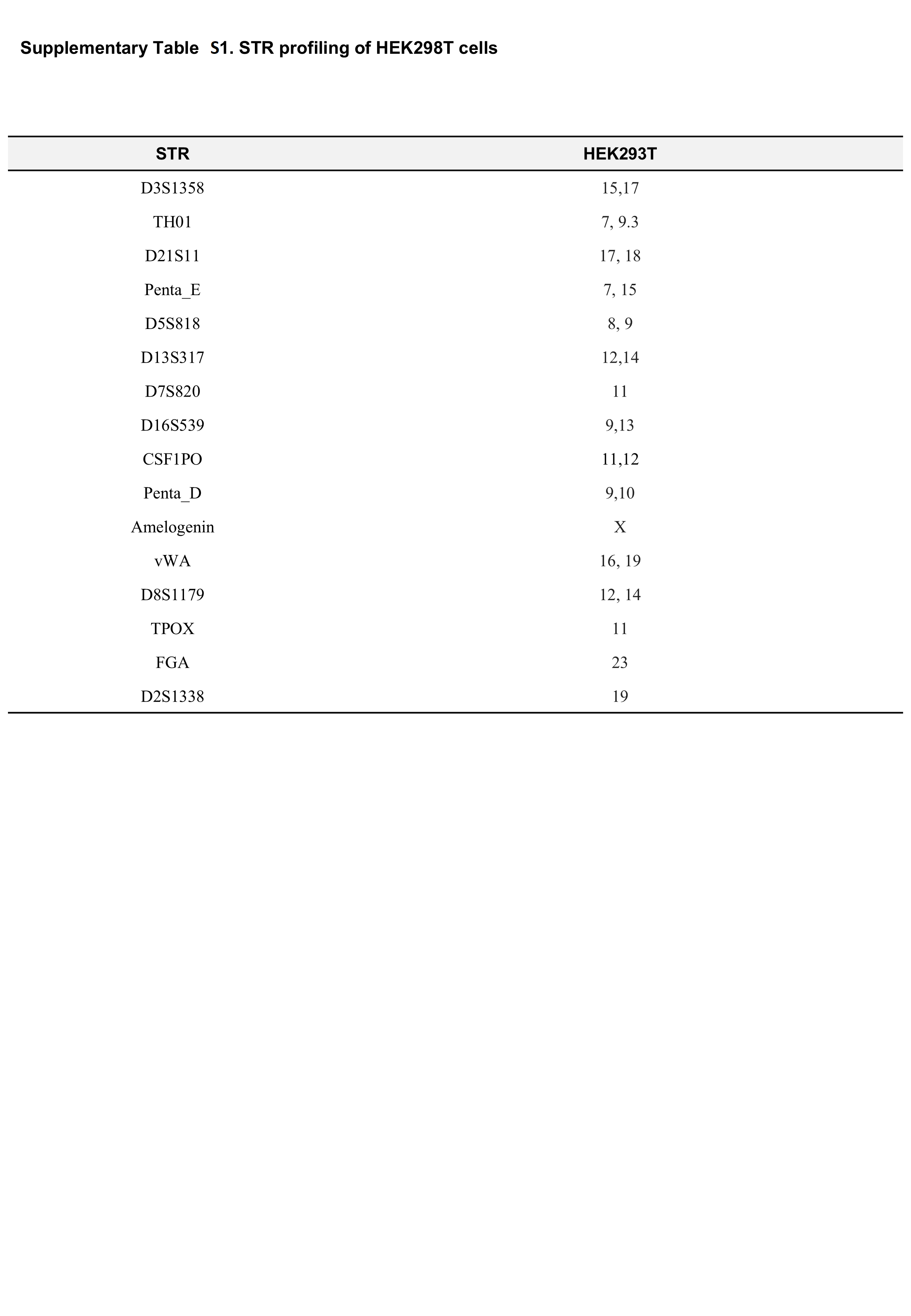

Supplement: Supplementary file 1 [file bioengineering-12-00409-s001.zip › Supple Table S1.TIF]

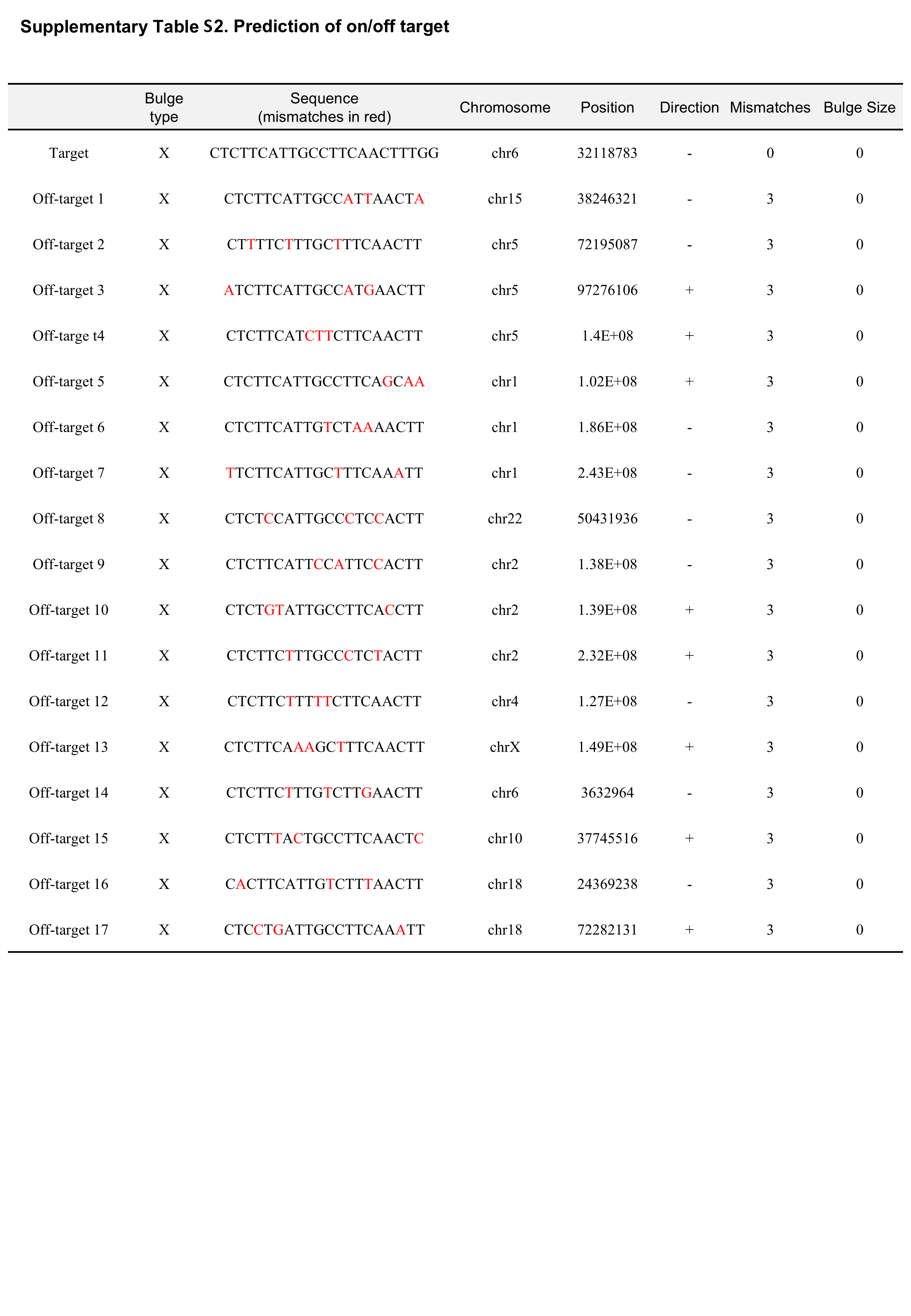

Supplement: Supplementary file 1 [file bioengineering-12-00409-s001.zip › Supple Table S2.TIF]

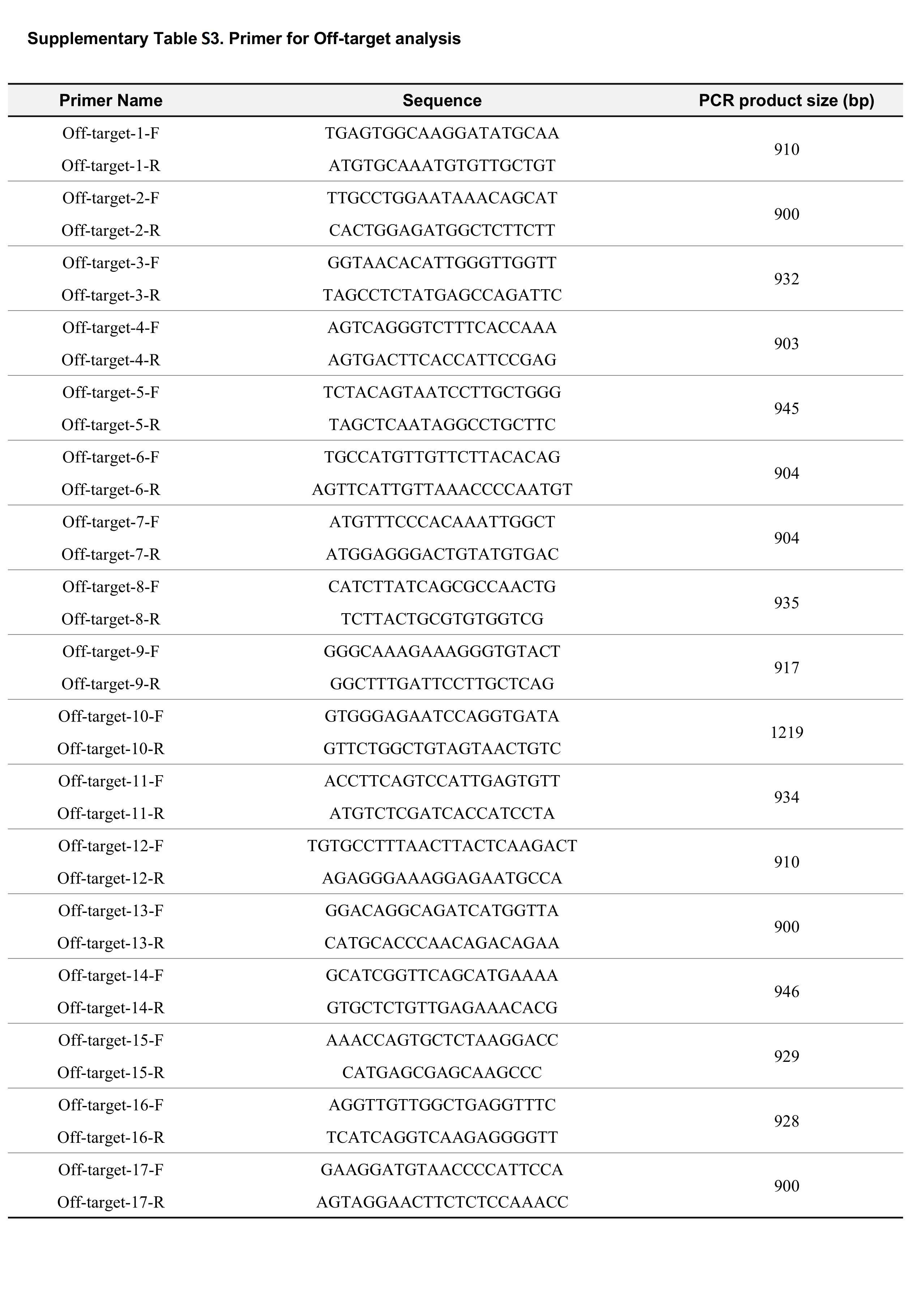

Supplement: Supplementary file 1 [file bioengineering-12-00409-s001.zip › Supple Table S3.TIF]

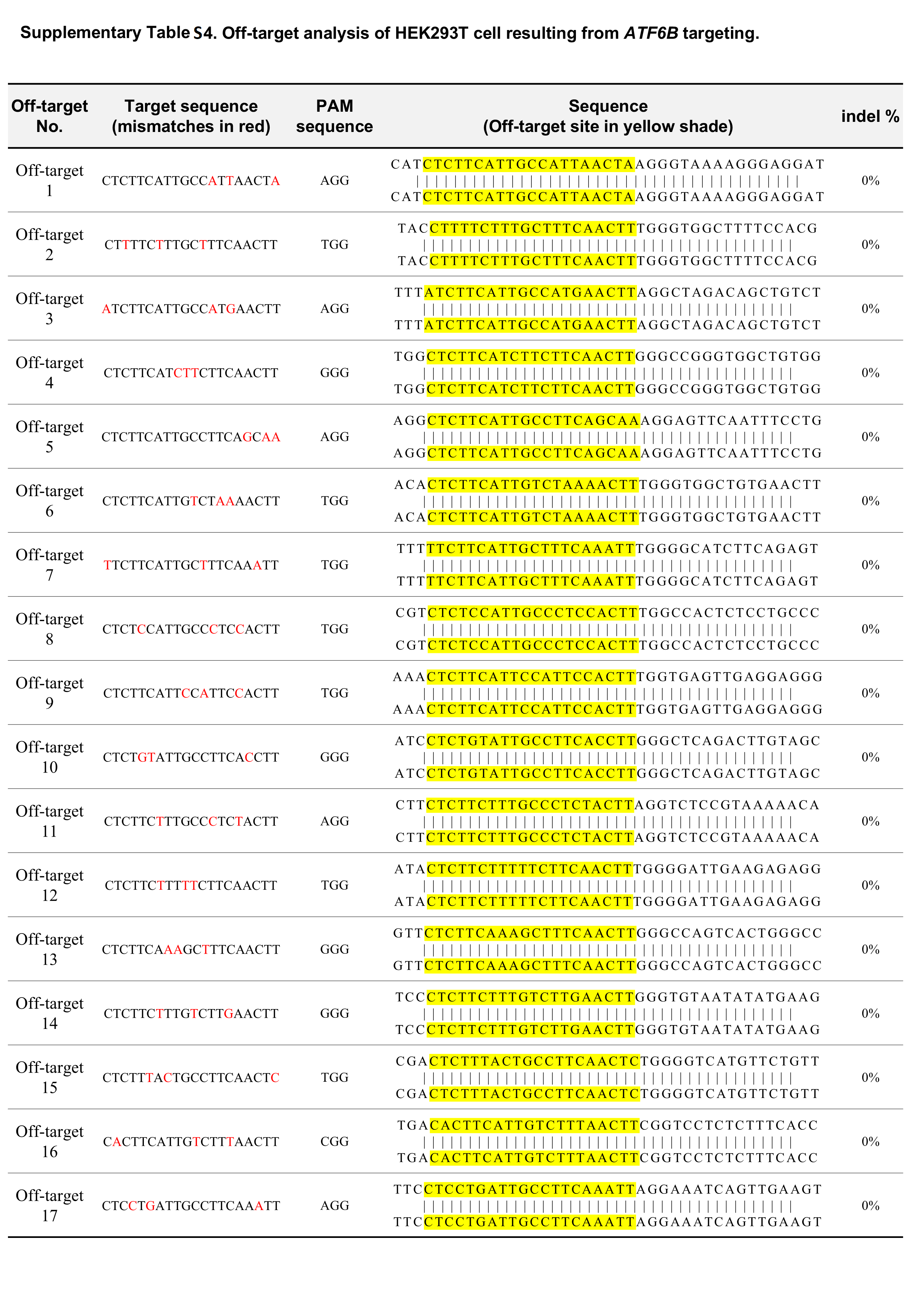

Supplement: Supplementary file 1 [file bioengineering-12-00409-s001.zip › Supple Table S4.TIF]
